# Fabrication of magnesium oxide–calcium alginate hydrogel for scaffolding yttrium and neodymium from aqueous solutions

**DOI:** 10.1038/s41598-023-42342-4

**Published:** 2023-09-23

**Authors:** M. Ghaly, B. A. Masry, E. M. Abu Elgoud

**Affiliations:** Hot Laboratories and Waste Management Center, Egyptian Atomic Energy Authority, 13759 Inshas, Egypt

**Keywords:** Chemistry, Engineering, Materials science, Nanoscience and technology

## Abstract

In this research, the possibility of using sustainable nano-MgO/Ca-alginate beads for efficient sorption of some rare earth metal ions such as neodymium(III) and yttrium(III) from an aqueous acidic solution was explored. The nano-MgO/Ca-alginate beads adsorbent was characterized before and after sorption of Nd(III) and Y(III) using scanning electron microscopy (SEM), Fourier-transform infrared spectroscopy (FT-IR), energy dispersive X-ray analysis (EDX), and X-ray diffraction (XRD) techniques. Batch sorption parameters were investigated, such as contact time, initial metal ion concentration, and adsorbent dose (V/m). The calculated experimental results showed that the suitable selected sorption conditions were carried out using 100 mg/L of Nd(III) and Y(III) with nano MgO/Ca-alginate beads (contact time = 90 min, pH = 2, V/m = 0.05 L/g). The maximum sorption capacity of 0.1 g of nano MgO/Ca-alginate was found to be 7.85 mg/g and 5.60 mg/g for Nd(III) and Y(III), respectively. The desorption of Nd(III) and Y(III) from the loaded nano MgO/Ca-alginate was achieved with 1.0 M sulfamic acid and found to be 51.0% and 44.2%, respectively. The calculated thermodynamic parameters for the nano MgO/Ca-alginate/Nd/Y system show that the positive charge of ΔH^o^ confirmed the endothermic nature of the sorption process, ΔS^o^ (positive) indicates an increase in reaction system disordering, and ΔG^o^ (negative) indicates a spontaneous process. These kinetic results indicate that the sorption process of Nd(III) and Y(III) on nano MgO/Ca-alginate beads is performed by the chemisorption process.

## Introduction

Rare earth elements (REEs) are a group with many different properties and levels in the environment. The uses, applications, and demand for REEs have expanded over the years. Because of their unique properties, REEs are widely applied in chemical engineering, the nuclear industry, metallurgy, medicine, electronics, and computer technology. Globally, most REEs are used for catalysts and magnets^1^. REEs are expected to be released and found in wide-spectrum concentrations in the environment and in waste materials. In order to address the increasing demands for REEs, effective separation and recovery are necessary. In this regard, recycling rare-earth element-containing products as well as their recovery from wastewater is quite important. It is worth highlighting that the separation of rare earth metal ions can be both complex and challenging owing to their similar properties.

Several approaches, such as extraction^2,3^, adsorption^4–9^, precipitation, flotation, membrane, and ion exchange, have been proposed for the pre-concentration and separation of REEs from aqueous solutions. Adsorption is the most effective and has been most frequently investigated for this purpose because it is highly efficient and convenient to handle. The recovery of lanthanides or valuable metals from water or wastewater can often result in considerable cost savings and have both ecological and economic benefits^10,11^. Owing to the reversible nature of most adsorption processes, the adsorbents can be regenerated by suitable desorption processes for multiple uses. Neodymium has become increasingly significant as an efficient material for permanent magnets used in hard drives, automotive generator motors, and automotive^6^. Various significant implementations for neodymium include the production of lasers for utilization in medicine and dentistry, rubber, superalloys, and advanced ceramics. Yttrium is widely utilized in the production of a variety of advanced products, including temperature sensors, color televisions, computer monitors, and microwave communication for the satellite industry. Yttrium-90 has some medical applications, such as the treatment of some diseases like liver cancer. Moreover, due to the importance of neodymium and yttrium as fission products in radioactive waste, the adsorption of these metals has been the subject of several investigations on different adsorbents. As a result, efforts to develop efficient techniques for recovering neodymium and yttrium from fission products and wastewater are ongoing. There have been various efforts to use the adsorption method to recover several REEs from the aqueous solution. Zhao et al.^12^ employed graphene oxide-tris(4-aminophenyl) amine composites for the adsorption of some REEs from aqueous solutions They reported that the maximum adsorption capacities for Y(III), Nd(III), Er(III), La(III), and Yb(III) are 10.52, 20.60, 26.52, 11.24, and 30.88 mg/g, respectively. The sorption of some REEs by using a chromium-based metal–organic framework (MIL-101-PMIDA) has been investigated by Lee et al.^13^.

Their results indicated that the MIL-101-PMIDA possesses maximum adsorption capacities of 70.90, 37.4, 49.0, 72.7, and 90.0 mg/g for Nd(III), La(III), Ce(III), Sm(III), and Gd(III), respectively. Adsorption of some rare earth ions has been studied using graphene oxide nanosheets by Ashour et al.^14^. They found high adsorption capacities of 85.67, 188.6, 225.5, and 135.7 mg/g for La(III), Nd(III), Gd(III), and Y(III), respectively. Saha et al.^15^ synthesized the phosphorous functionalized nanoporous carbon for the recovery of Nd(III) and Dy(III). They found that the synthesized composite can achieve maximum adsorption capacity for Nd(III) (335.5 mg/g) and Dy(III) (344.6 mg/g). Adsorption of Y(III) from an aqueous solution using titanium dioxide with surface arsenate groups (4As–TiO_2_) and (Nd/4As–TiO_2_) has been examined by Vasylyeva et al.^16^. Their results indicated that the experimental adsorption capacities of Y(III) are 24.8 and 127.0 mg/g by using Nd/4As–TiO_2_ and 4As–TiO_2_, respectively. Sakr et al.^17^ evaluated the behavior of 3-Amino-5-hydroxypyrazole impregnated bleaching clay (AHIBC) for the sorption of Y(III). Their study revealed that the AHIBC has a maximum Y(III) adsorption capacity of 171.32 mg/g. The recovery of Nd(III) from aqueous solutions was performed by De Vargas Brião et al.^18^ using expanded vermiculite. According to their findings, Nd(III) has a maximum adsorption capacity of 69.24 mg/g. Lima et al.^19^ employed two strains of Spirulina platensis (LEB-18 and LEB-52) for the recovery of Nd(III) from aqueous solutions. They reported that LEB-18 possesses a maximum biosorption capacity of 72.5 mg/g and LEB-52 has a maximum biosorption capacity of 48.2 mg/g. Chitosan-Manganese-Ferrite Magnetic Beads (CS-MF) have been studied by Durán et al.^20^ for their ability to adsorb Nd(III) from the aqueous phase. The resulting magnetic CS-MF beads have a maximum adsorption capacity of 44.29 mg/g. Alginate-based adsorbents have been used to separate and remove metal ions in numerous reports^21–23^. For the adsorption of REEs, Wang et al.^24^ synthesized calcium alginate-poly glutamic acid hybrid gels (ALG-PGA). According to their results, the ALG-PGA has a maximum Nd(III) adsorption capability of 1.65 mmol g^−1^. Fila et al.^25^ investigated the adsorption of some REEs on alginate-lignin composite (ALG-L) from aqueous solutions. Their research revealed that the ALG-L composite has adsorption capabilities of 109.56, 97.97, 97.98, and 98.68 mg/g for La(III), Ce(III), Pr(III), and Nd(III), respectively. Fila et al.^26^ synthesized biochar-doped and clinoptilolite-doped sodium alginate (ALG5-BC1 and ALG5-CPL1) biocomposites for the sorption of some lanthanide elements. Their work revealed that ALG5-BC1 and ALG5-CPL1 have Nd(III) adsorption capacities of 108.88 and 102.70 mg/g, respectively. Recently, the efforts of researchers have focused on finding effective adsorbents that may be used in the recovery of rare earth elements. Alginate is an extracellular biopolymer derived mainly from brown seaweed and different microorganisms as well as it consists of chains of 1,4-linked-D-mannuronic acid and -L-guluronic acid^27,28^. Furthermore, it is one of the most significant natural polysaccharides, with an abundance of carboxyl and hydroxyl groups. Additionally, when carboxyl groups interact with high valent metal ions (for example, Ca^2+^), a hydrogel microcapsule is created. Many studies have been reported on the separation and removal of metal ions using alginate-based adsorbents^29,30^. Furthermore, cross-linked alginate gel is a promising host matrix for ionizing agents because it allows them to easily form capsules^31–38^.

The significance of choosing combine mixture of Nd and Y for adsorption study is to investigate the feasibility of sorption and separation between light and heavy lanthanides. Where Nd represents light rare earth and Y represent one of heavy rare earths metal ions. On the other hand the two metal ions were in different applications such as Neodymium-stabilized yttrium aluminum garnet (YAG) which is the main component of many modern lasers.

As well as the novelty of nano-MgO/Ca-alginate composite lies in its unique combination of materials and potential applications. The composite material combines the properties of MgO, known for its excellent adsorption capabilities and high surface area with alginate, a biopolymer derived from seaweed that offers Eco-friendliness and biocompatibility This composite could exhibit enhanced sorption capabilities compared to individual components, making it suitable for various applications, such as wastewater treatment, separation of rare earth elements, heavy metal removal, and other environmental remediation processes. Its novelty lies in the synergistic effects of the two materials, which may lead to improved sorption efficiency and selectivity for specific contaminants.

In this investigation, the magnesium oxide-alginate hydrogel was fabricated using magnesium oxide and sodium alginate as a matrix and directed to study the sorption of neodymium and yttrium from acidic nitrate solution using the strongly acidic cationic exchange resin nano MgO/Ca-alginate beads using batch technique. The effects of different parameters on the sorption and separation processes will be investigated, such as contact time, nitric acid concentration, V/m ratio, and temperature. Desorption investigations will also be carried out and evaluated. Separation feasibility between the investigated REEs is also discussed based on the difference between their sorption and desorption behavior.

## Experimental

### Materials and chemicals

The chemicals used in this work were of analytical reagent grade (AR), and most of them were used without further purification. Calcium chloride (Fluka), Magnesium sulfate (El-Naser pharmaceutical company), sodium alginate, boric acid (Sigma-Aldrich), and ammonia solution (Merck), stock solutions of Nd(III) and Y(III) (1000 mg/L), were prepared by dissolving a known amount of the metal oxide in minimum concentrated nitric acid, evaporated to near dryness, and then made up to the mark in a measuring flask with double-distilled water. Desorption reagents such as sulfamic acid, nitric acid, sulfuric acid, ammonium carbonate, and sodium acetate were purchased from Pubchem.

### Preparation of Nano-MgO

Magnesium hydroxide was used as a precursor material for the preparation of nano-MgO. The precipitation method was used for the preparation of nano-Mg(OH)_2_ at an ambient temperature of 333 K. Ammonia solution was dropwisely added to 2 M magnesium sulfate solution under vigorous stirring at 3000 rpm, then ceased when the concentration of H^+^ reached 10.5. The suspension was aged for 2 h before being centrifuged for 2 min at 8000 rpm, and then washed three times with deionized water^39^. The collected precipitate was dried at 80 °C for 2.0 h to produce Mg(OH)_2_. Finally, the prepared Mg(OH)_2_ was calcinated at 550 °C to produce MgO nanoparticles.

### Preparation of nano-MgO/calcium alginate

**Figure 1:**
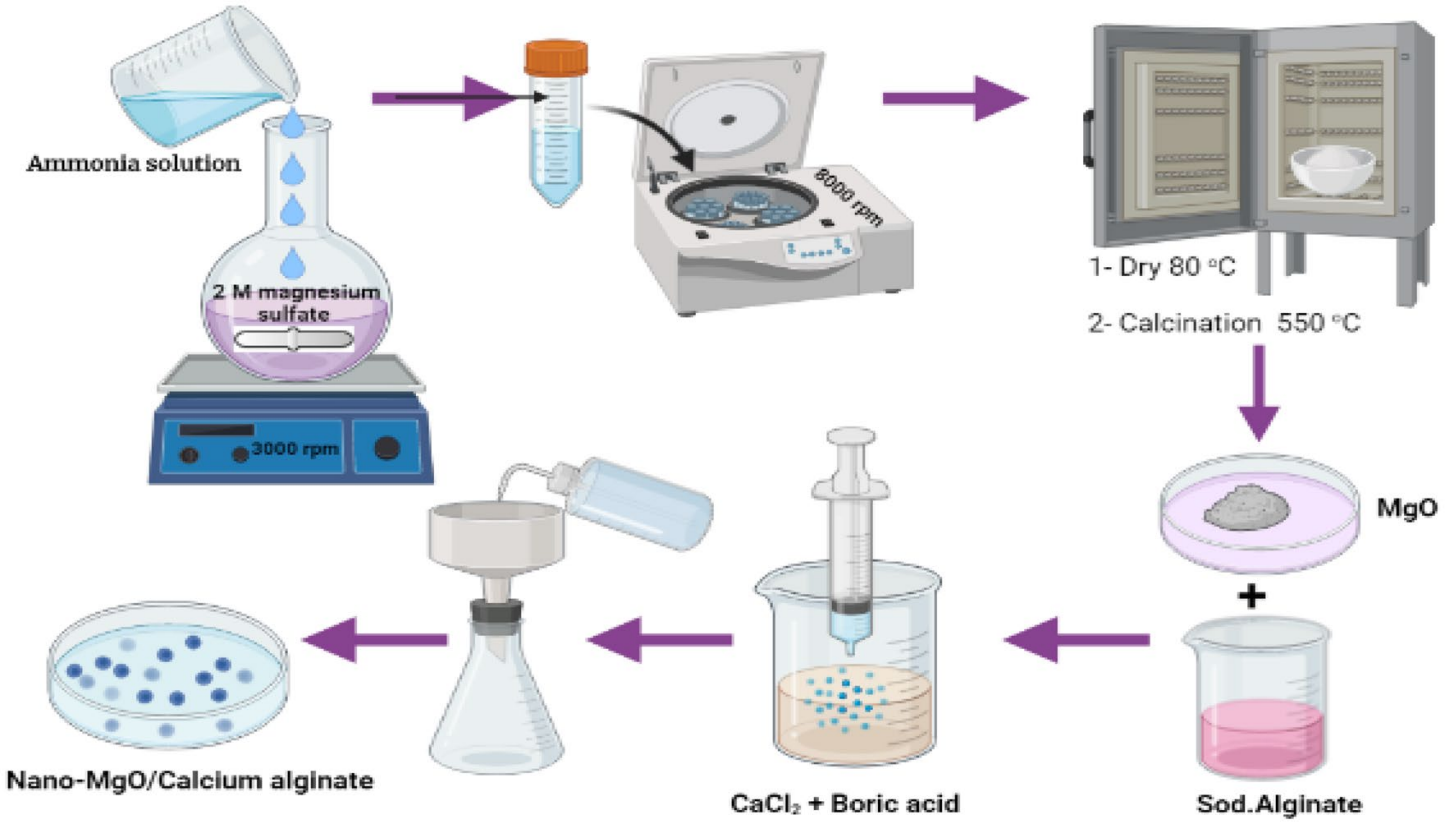
Preparation procedures of MgO/Ca-alginate beads.

To produce nano-MgO/Calcium alginate beads, the method reported by^39^ was followed with some modifications. Typically, 2 g of MgO nanoparticles and 10 g of sodium alginate were added to 100 mL of double-distilled water under stirring for 4.0 h at room temperature. The formed suspension was then dropwisely added to a saturated costing solution of boric acid and CaCl_2_. The formed beads were kept in the costing solution for 2.0 h to confirm the formation of stabilized spherical capsules. Finally, the formed capsules were washed many times with double-distilled water and then stored for further usage. The schematic representation for the preparation of MgO/Ca-alginate beads is given in Fig. 1.

### Sorption batch experiments

The sorption experiments were carried out under the following conditions: V/m = 0.05 L/g, Nd(III) and Y(III) concentrations = 100.0 mg/L in aqueous acidic solution of pH 2. In each adsorption experiment, 5 mL of the investigated metal ion solution was added to 0.1 g of MgO/Ca-alginate nano-beads in stoppered glass bottles, which were then shaken at 25 ± 1 °C in a water thermostatic shaker. The concentrations of Nd(III) and Y(III) ions were measured using a UV–visible spectrophotometer (a Shimadzu UV-160, Japan) with the Arsenazo(III) method^40^.

The sorption percentage (S%) at equilibrium was calculated from the Eq.^41^:

1 $$S\% \, = \,\tfrac{{C_{i} - C_{e} }}{{C_{i} }} \times 100$$

The adsorption capacity (q_e_) at equilibrium was given by the Eq.^42^:

2 $$q_{e} = (C_{i} - C_{e} ) \times \left( {\tfrac{V}{m}} \right)$$

where and are the initial and equilibrium metal ion concentrations (mg/L) of metal ions, respectively; V is the volume of the used aqueous solution in liters (L); and w is the weight of the adsorbent (g).

On the other hand, the desorption stage of the metal ions under inquiry was examined utilizing several stripping reagents, including mineral acids, sodium carbonate, ammonium carbonate, and citric acid. In this case, under the identical sorption testing conditions, 0.1 g of nano-MgO/Ca-alginate beads were shaken with 5.0 mL of the stripping solution for 90 min. The nano-MgO/Ca-alginate beads were loaded with approximately 100.0 mg/L of each individual Nd(III) and Y(III).

### Nano-MgO/Ca-alginate beads characterizations

Visualization of nano-MgO/Ca.alginate morphology was performed using a scanning electron microscope (SEM) with Energy Dispersive X-ray analysis (EDX; ZEISS-Evo 15-UK). Structure determinations were carried out using X-Ray Diffraction (XRD, Schimadzo X-ray diffractometer) and confirmed using Fourier Transform infrared (FTIR, PerkinElmer, BX spectrometer), where the range of frequency is 4000–400 cm^−1^.

## Results and discussion

### Characterization of nano-MgO/Ca-alginate beads

#### XRD analysis

**Figure 2:**
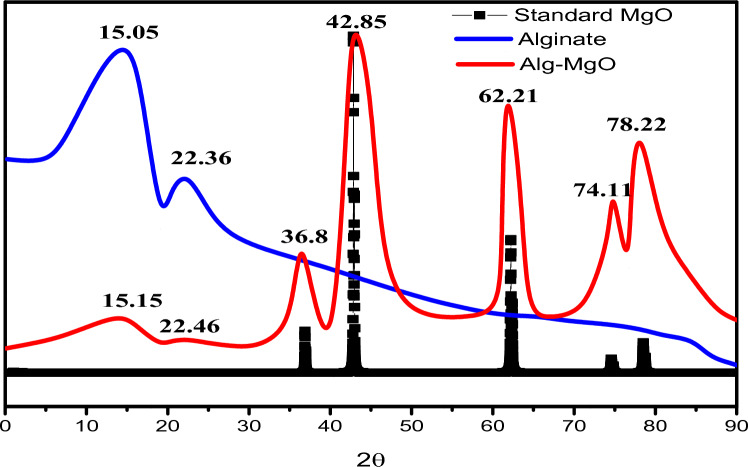
X-ray diffraction patterns of Standard MgO, Ca-alginate and Alg-MgO composite.

The XRD pattern of Alg-MgO is depicted in Fig. 2. The main characteristic peaks of alginate appeared at the angular positions of 15.05 and 22.36. On the other hand, the observed peaks that were placed at 2θ = 36.8, 42.85, 62.21, 74.11 and 78.22 are in excellent agreement with the standard crystalline structure of periclase MgO, (COD-1011116). The existence of all these peaks confirms the successful addition of MgO to ca-alginate to form Alg–MgO composite without any effect on the structure of the polymer blend^43,44^.

The crystalline size of MgO has been estimated using Debye–Scherer’s formula^45^:

3 $$D = \frac{{K{\uplambda }}}{\beta Cos\theta }$$

where K is the shape factor (0.9), λ: is the wavelength of (Cu-Kα1 radiation ) that used in X-ray diffraction analysis (λ = 0.15406 nm), β and θ express the full width at half maximum(FWHM) and the angular positon of the diffraction peak, respectively. Using this formula the average crystalline size of MgO that encapsulated inside the bead was found to be 25.0 nm.

#### Fourier transform infrared (FTIR) analysis

**Figure 3:**
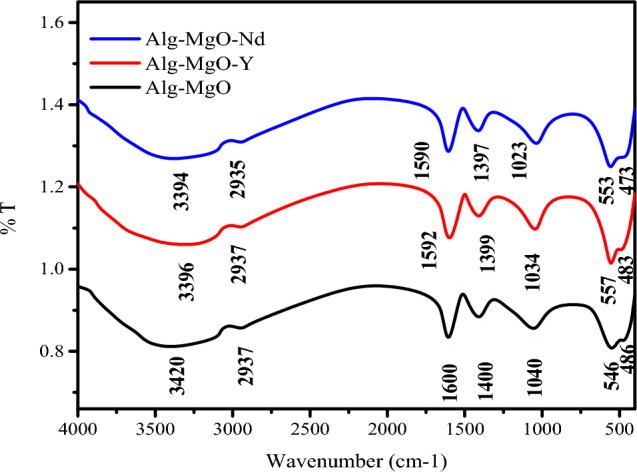
FT-IR spectra of nano-MgO/Ca-alginate beads before and after sorption of Nd(II) and Y(II) from acidic nitrate medium at pH = 2.

A plot of transmittance (T%) versus wavenumber (cm^−1^) of Alg–MgO and Alg–MgO–Nd, and Alg–MgO–Y is shown in Fig. 3 and used for the purpose of comparison. The FTIR spectrum showed the appearance of significant peaks in the major function groups of alginates. The peaks located at 3420–3394 cm^−1^^46,47^ were attributed to hydroxyl stretching vibrations of alginate, while those that appeared around (2937–2935) cm^−1^ were due to aliphatic C–H Stretching vibrations. The asymmetric and symmetric vibrations of carboxyl were found at about (1600–1590) cm^−1^ and (1400–1997) cm^−1^, respectively. Peaks that exist at (1040–1023) cm^−1^ correspond to C–C functional groups^48,49^. The main two characteristic peaks of magnesium oxide appeared at about (540–553) cm^−1^ and (486–473) cm^−1^ where the slight shift that occurred in all the above peaks is attributed to sorption of Y and Nd into the Alg–MgO composite. This legally supports the suggested sorption mechanism in "Suggested sorption mechanism of Nd and Y with nano-Mgo/Ca-alginate" section.

#### Nano-MgO/calcium alginate morphology

**Figure 4:**
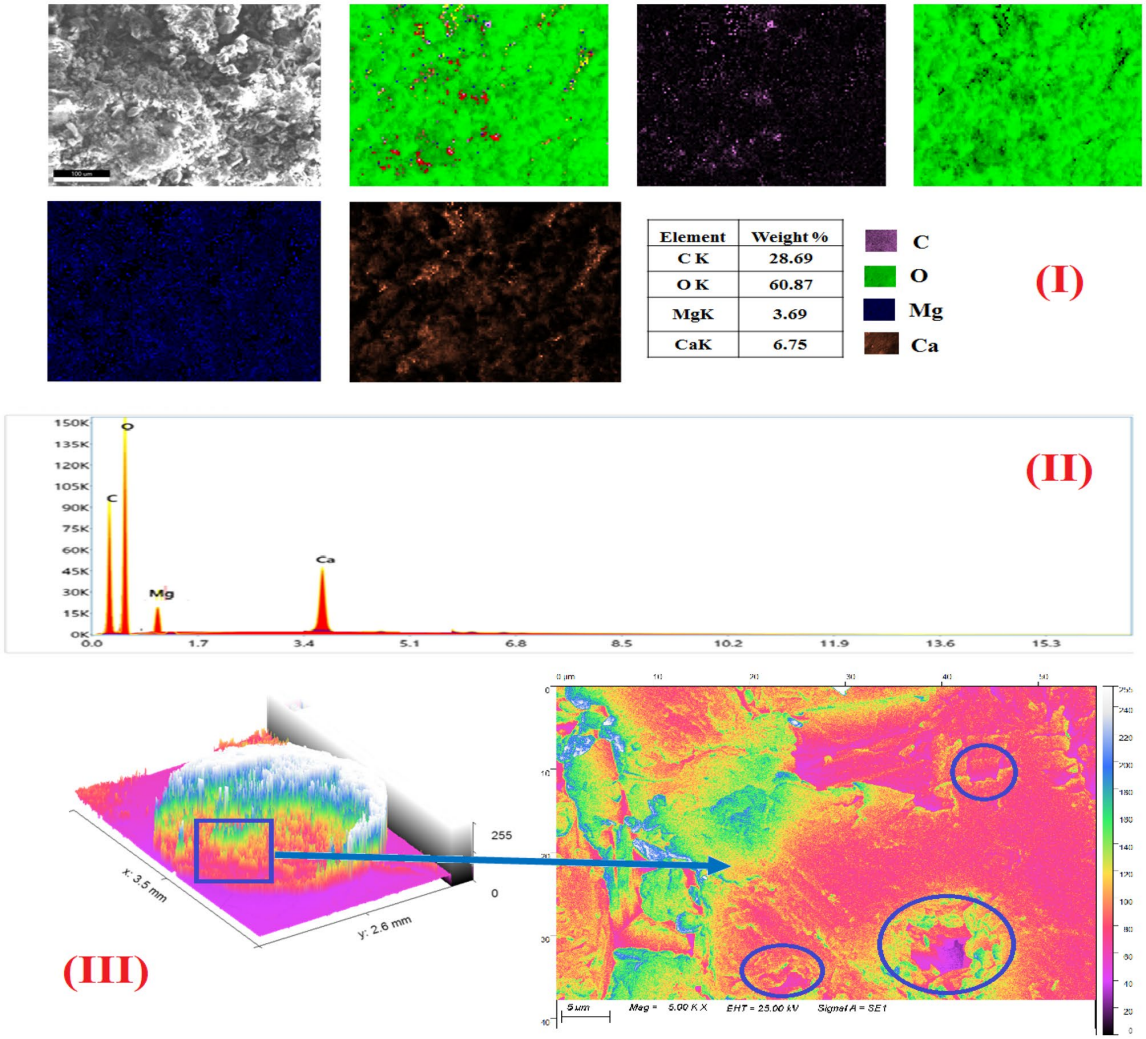

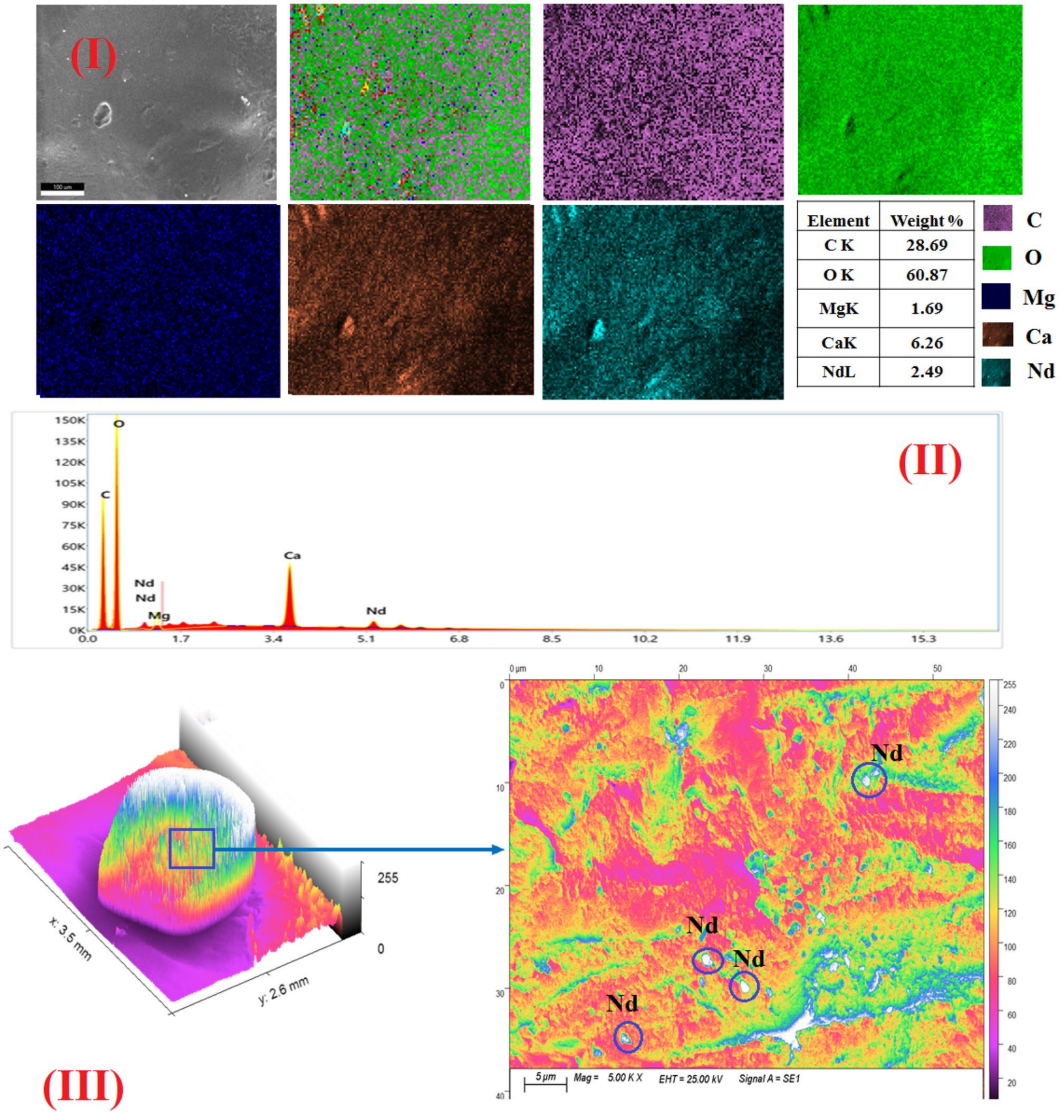

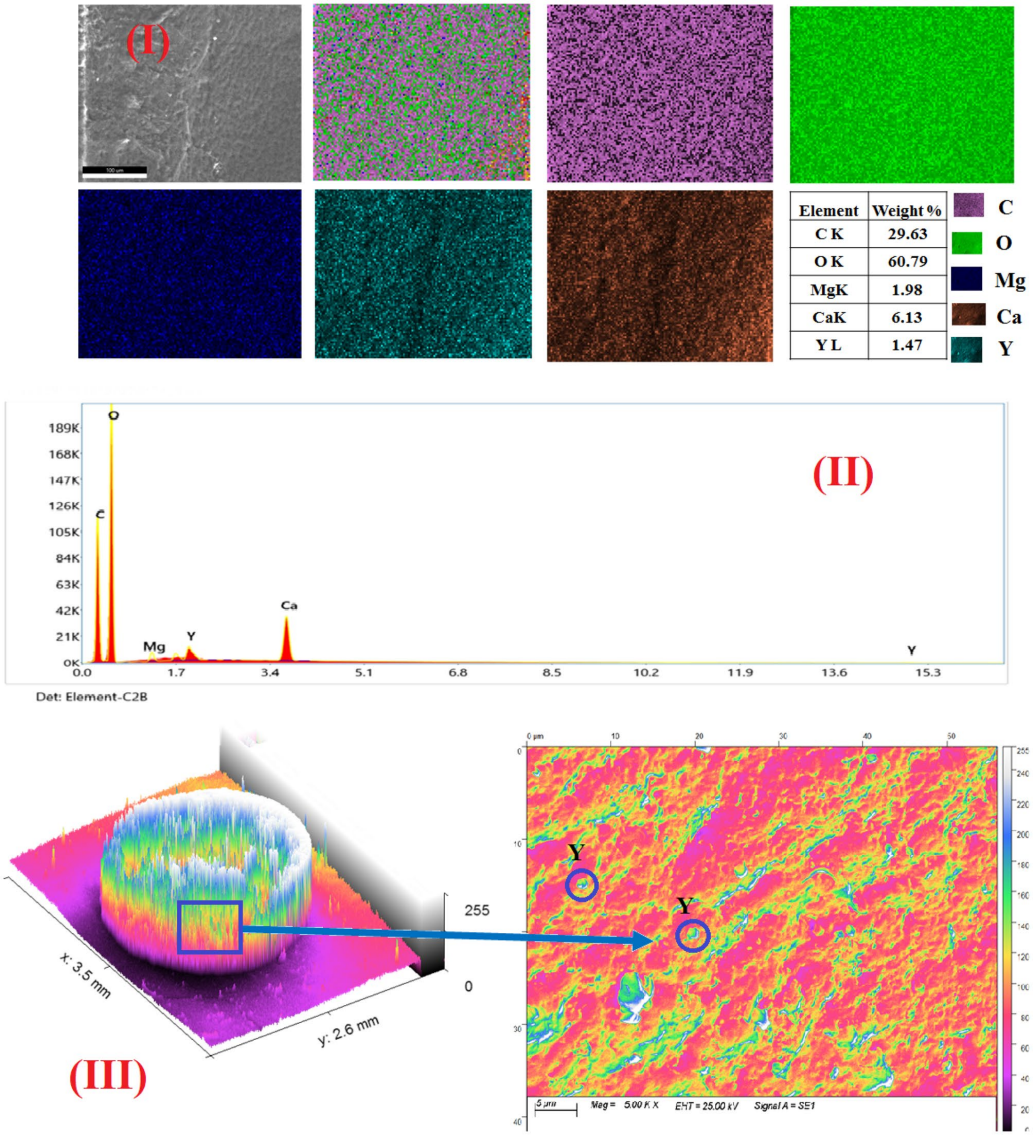
(**a**) The surface morphology of nano-MgO/Ca-Alg hydrogel bead (I) SEM images (II) EXD- analysis (III) 3D- colored images at low and high magnification before sorption process. (**b**) The surface morphology of nano-MgO/Ca-Alg hydrogel bead (I) SEM images (II) EXD-analysis (III) 3D- colored images at low and high magnification after sorption process of Nd at pH = 2. (**c**) The surface morphology of nano-MgO/Ca-Alg hydrogel bead (I) SEM images (II) EXD-analysis (III) 3D-colored images at low and high magnification after sorption process of Y at pH = 2.

The surface morphology of nano-MgO/Ca-Alg was performed by SEM, which is depicted in Fig. 4a, b, c at a magnification of 1.5 k. SEM images indicate that MgO/Ca-Alg has a rough morphology with a large number of openings, pores, and small slits. Mapping images confirm the successful uniform insertion of MgO into the calcium alginate groove, which enhances the mechanical stability of Ca-Alg. The 3D-colored images of beads at low and high magnification confirm the existence of a large number of cracks and pores on the external surface of MgO/Ca-Alg which became filled with the sorbed metal ions Y^3+^/Nd^3+^. EDX-data of MgO/Ca-Alg hydrogel before the sorption process revealed the existence of Mg, Ca, O, and C, while additional peaks either of Nd or Y appeared after the sorption process. It is also noticed that the percentage of Mg decreases after sorption, which confirms its exchange with Y or Nd and support the suggested mechanism in "Suggested sorption mechanism of Nd and Y with nano-Mgo/Ca-alginate" section.

### Sorption batch experiments

#### Effect of shaking time

**Figure 5:**
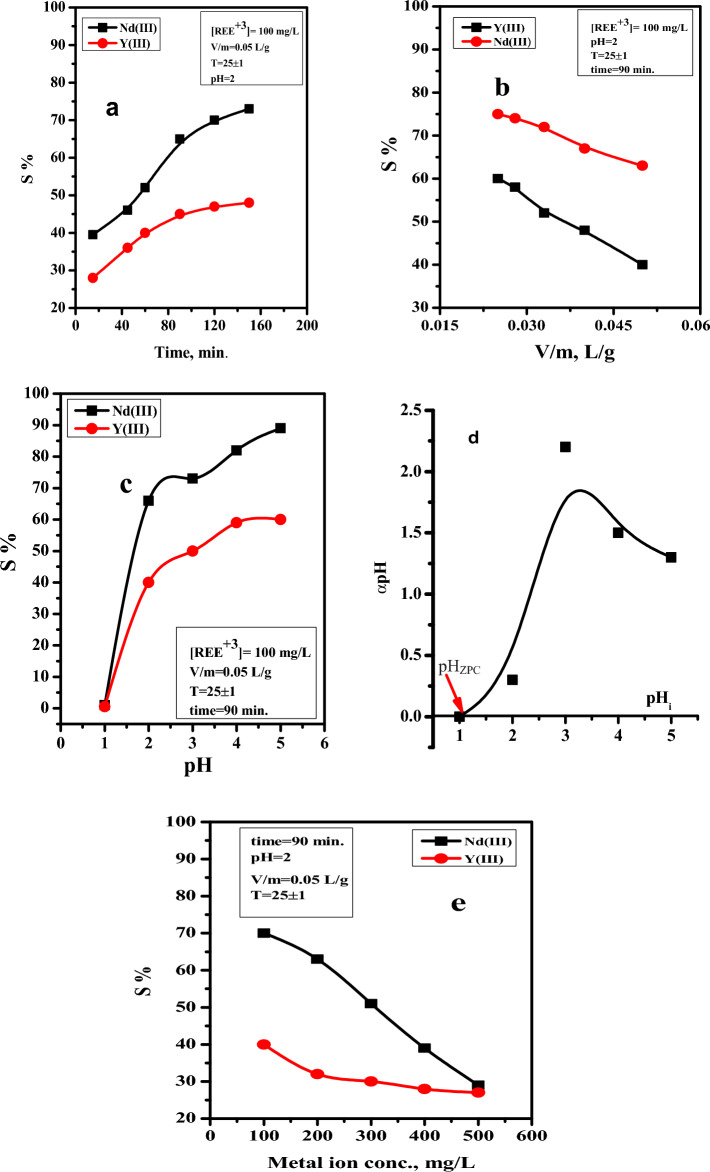
(**a**) Effect of contact time, (**b**) adsorbent dosage (V/m), (**c**) solution pH, (**d**) The zero of the point charge, (**e**) initial REEs^+3^ concentrations on the sorption percentages of Nd(III) and Y(III) using nano-Mgo/Cal-alginate beads from acidic nitrate medium.

Using nano MgO/Ca-alginate beads as an adsorbent from nitrate solution, the effect of contact duration on the sorption effectiveness of Nd(III) and Y(III) ions was investigated over the course of 1–160 min. As a result, the equilibrium contact time for the sorption of the investigated metal ions using nano-MgO/Ca-alginate beads was determined by the results, which showed that the rare earth ion sorption process took place between 1 and 90 min and was dependent on the presence of vacant active sites. The rate of sorption on the surface of the adsorbents was significantly constant at contact times beyond 90 min, as shown in Fig. 5a. This may be because of the available active sites on the sorbent surface. As a result, the nano-MgO/Ca-alginate bead adsorbent was used, and the equilibrium contact time for the sorption of the examined metal ions was set at 90 min.

#### Adsorbent dosage (V/m)

Another important factor that significantly influences the sorption process is the sorbent dosage, which can control how well the sorbent binds to the examined metal ions at a given initial concentration. In this regard, the influence of the sorbent dosage (V/m) on the Nd(III) and Y(III) ion sorption efficiency was examined in the range of 0.025–0.05 L/g, as shown in Fig. 5b. According to the data obtained under the chosen batch conditions, the sorption percentage yield follows the sequence Nd(III) > Y(III) and decreases for both metal ions as the V/m value increases. The chosen adsorbent dosage (V/m) of nano-MgO/Ca-alginate beads were set at 0.05 L/g in all studies based on the results, since this gave sensible sorption percent for Nd of (65%) and for Y of (40%).

#### Effect of solution pH on sorption of Nd and Y using nano MgO/Ca-alginate beads

The effect of solution pH on Nd and Y sorption using nano-MgO/Ca-alginate beads at optimized sorption conditions was investigated and is shown in Fig. 5c. The experimental results showed that the sorption efficiency of nano-MgO/Ca-alginate beads was more effective towards Nd than Y at the different pH levels tested. The calculated results show that the sorption efficiency of Nd and Y increased as the pH range increased from 2 to 5, as shown in Fig. 5c. However, the pH was set to 2 in all experimental studies as a suitable low-acid medium for capturing REEs, which give S% as (65% and 40%) for Nd and Y, respectively. The selection of pH = 2 is regarding working from a low acidic medium, closing to the fact that fission products were dissolved in a highly concentrated acid medium. As a result, the zero of the adsorbent's point charge is 1, as shown in Fig. 5d, and the adsorbent's surface active sites will be negatively charged when pH > 1, making them appropriate sites for the adsorption of the cationic species Nd^3+^ and Y^3+^, enhancing the adsorption process. However, at pH < 1, the adsorption efficiency is reduced.

#### Effect of metal ions concentration

The effect of initial Nd(III) and Y(III) concentrations on sorption percentage was investigated in the range of 100–500 mg/L using nano-MgO/Ca-alginate beads from an acidic nitrate medium with a pH of 2. The experiments were conducted by individually shaking 5.0 mL of the investigated metal ion solution with 0.1 g of the adsorbent for 90 min at 25 °C. The results are shown in Fig. 5e, where the sorption efficiency decreases as the concentration of REEs^+3^ increases in relation to saturation, which can be attributed to interactions between the adsorbent active sites and these metal ions. However, the working concentration of the selected metal ion is 100 mg/L. under the other selected sorption factors, gives S% of 65% and 40% for Nd and Y, respectively.

#### Suggested sorption mechanism of Nd and Y with nano-Mgo/Ca-alginate

**Figure 6:**
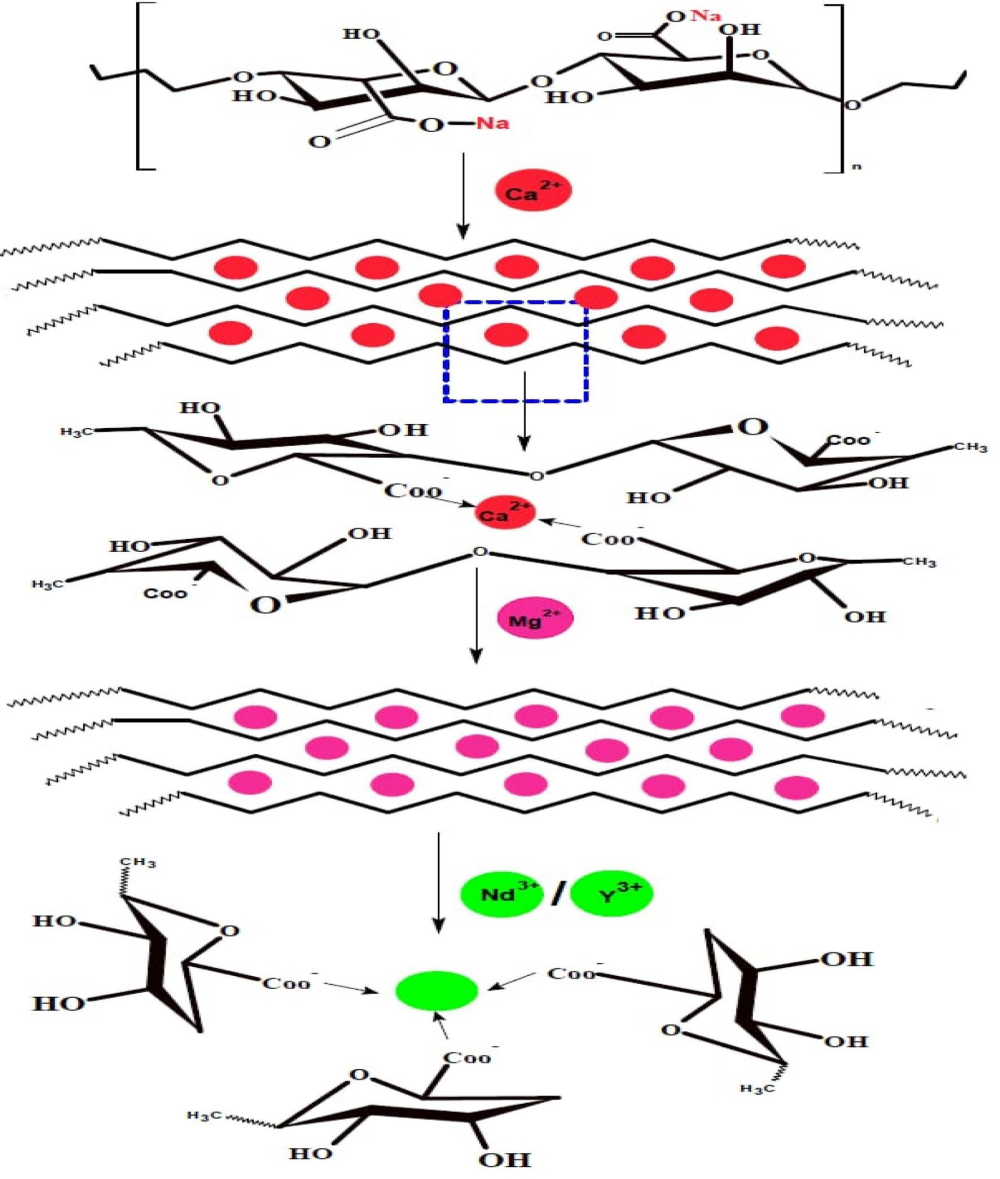
Schematic representation of the mechanism interaction between Nd and Y with nano-MgO/Ca-alginate.

Based on the experimental results and considering that M (NO_3_)_2_^+^ is the predominant species in pH = 2^27,43^, where M represents Nd(III) and Y(III), the ion exchange extraction mechanism of REEs metal ion (M) with MgO/Ca-alginate nano beads was suggested to proceed via reaction pathways as represented in Fig. 6. During the encapsulation of magnesium oxide inside alginate, the high-polar carboxylate groups of alginate act as coordination sites and attract Mg^2+^ and form nano-MgO/Ca-alginate bead^46^ and these procedures were confirmed in the "Fourier transform infrared (FTIR) analysis" and "Effect of solution pH on sorption of Nd and Y using nano MgO/Ca-alginate beads" sections.

#### Thermodynamic parameters and temperature effect

**Figure 7:**
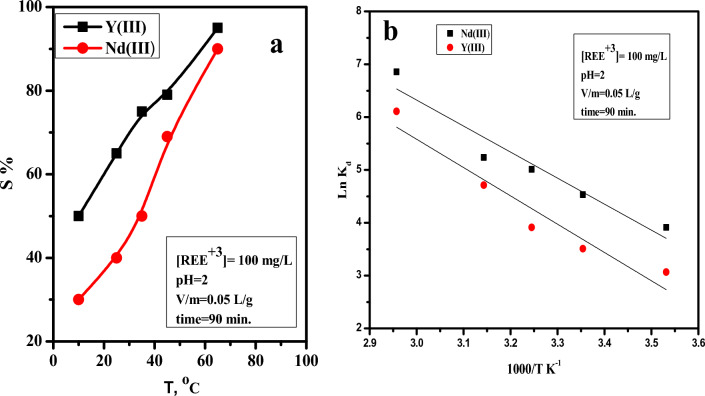
(**a**) The effect of temperature on the sorption of Nd and Y using nano-MgO/Ca-alginate beads from low acidic solution, (**b**) relation between 1/T and log K_d_ for the sorption of Nd and Y using nano-MgO/Ca-alginate beads from low acidic solution.

The effect of temperature ranging from 10 to 65 °C on the batch sorption of 100 mg/L of Nd(III) and Y(III) from acidic nitrate solution at pH = 2 using nano-MgO/Ca-alginate beads was investigated. The results plotted in Fig. 7a show that increasing temperature increased sorption efficiency, which could be attributed to an increase in the elasticity of the prepared MgO/Ca-alginate, which causes changes in the adsorbent active sites.

The thermodynamic parameters include the standard free energy change ΔG^o^, standard enthalpy change ΔH^o^, and the standard entropy change ΔS^o^ the sorption process can be calculated from the linear plot of 1/T vis log K_d_, shown in Fig. 7b, and was calculated by the following Eq.^50,51^.

4 $$\ln Kd \, = \frac{{\Delta S^{o} }}{R} - \frac{{\Delta H^{o} }}{RT}$$

And the free energy change (ΔG) was given Eq. (4):

5 $$\Delta {\text{G}}^{{\text{o}}} \, = \, \Delta {\text{H}}^{{\text{o}}} - {\text{T}}\Delta {\text{S}}^{{\text{o}}}$$

T is the Kelvin temperature (K), R is the gas constant (8.314 J/mol K), and k_d_ is the distribution coefficient (mL/g). The reported thermodynamic parameters are shown in Table 1; ΔH indicates the endothermic nature of the sorption process, ΔS indicates the increase in disordering of the reaction system, and ΔG indicates that the reaction is a spontaneous process. Regarding to that the enthalpy change values in the reported range (20.9–418.4 kJ/mol) as support chemisorption reaction^52,53^, and comparing with the obtained values which are 40.7 kJ/mol and 44.9 kJ/mol for Nd and Y respectively, confirming that the sorption of the investigated metal ions onto nano-MgO/Ca-alginate are chemisorption process. These achieved findings support the obtained results in the kinetics isotherm models.

**Table 1:** Thermodynamic parameters for Nd(III)/Y(III)-MgO/Ca-alginate beads from low acidic solution.

Metal ions		T, (K)		R^2^		ΔG^o^, (k J mole^−1^)		ΔH^o^, (k J mole^−1^)	ΔS^o^, (k J mole^−1^)
Nd(III)		293		0.915		− 10.7		40.7	175.4
		303				− 12.4			
		313				− 14.2			
		323				− 15.9			
		333				− 17.7			
Y(III)		293		0.914		− 8.0		44.9	180.4
		303				− 9.8			
		313				− 11.6			
		323				− 13.4			
		333				− 15.2			

### Sorption kinetics

**Figure 8:**
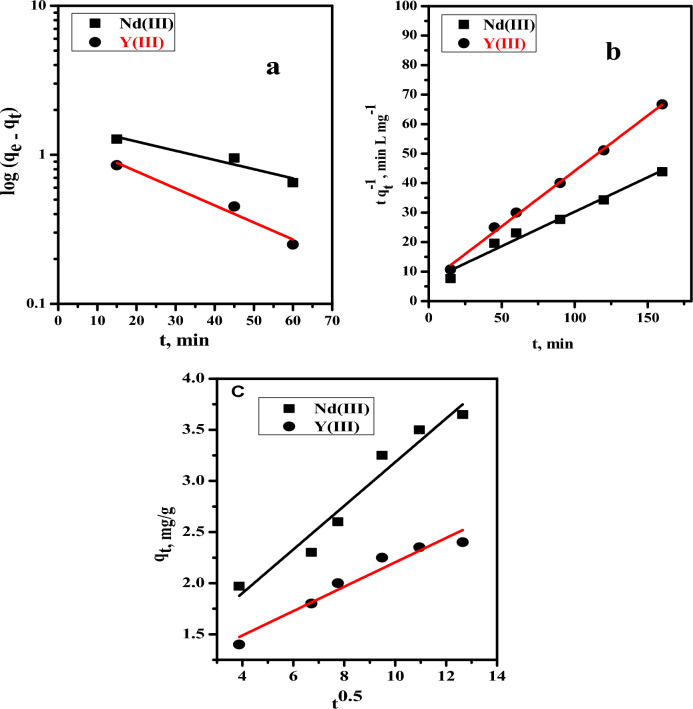
(**a**) First order kinetics model, (**b**) second order kinetics model, (**c**) Weber–Morris intra-particle diffusion model for sorption of Nd(III) and Y(III) on nano-MgO/Ca-alginate beads from low acidic solution.

The sorption kinetics of investigated metal ions on nano-MgO/Ca-alginate beads were studied using two well-known kinetic models: pseudo-first-order, pseudo-second-order and Weber–Morris intra-particle diffusion model Figs. 8a–c shows the plots of the two kinetic models. Table 2 summarizes the calculated values of correlation coefficients (R^2^) and the kinetic constants. It was found that the pseudo-second-order model possessed a higher R^2^ = 0.971 for Nd(III) and 0.996 for Y(III). These results show that the sorption process of Nd(III) and Y(III) on nano-MgO/Ca-alginate beads is performed by the chemisorption process. The values of k_ad_ obtained from the slopes of the two straight lines for Weber-Morris intra-particle diffusion are 0.214 and 0.119 mg/g min^1/2^ for Nd(III) and Y(III) ions, respectively, while the values of the intercept C are 1.05 and 1.012 mg/g. The correlation coefficients R^2^, which are very far from unity, indicate that this model is inapplicable for Nd(III) and Y(III) ions, and so the intra-particle diffusion model is not the rate governing step for the sorption of both metal ions onto nano-MgO/Ca-alginate beads. The smaller the value of the intercept C suggests, the thinner the boundary layer^54^. Furthermore, the deviation of the straight lines from the origin may be regarded to the difference in mass transfer rates in the early and final steps of the process^55^.

**Table 2:** Kinetic model parameters for adsorption of Nd(III) and Y(III) ([M^3+^] = 100 mg L^−1^, Dose = 0.10 g, V = 5.0 mL, pH = 2.0, T = 25 °C) from low acidic solution using nano-MgO/Ca-alginate beads.

Kinetic	Models	Parameters	Metal ions	
			Nd(III)	Y(III)
Pseudo-First order	$\mathrm{log}{(q}_{e}-{q}_{t})=\mathrm{log}{q}_{e}-\frac{{K}_{2 }}{2.303}t$	q_e_ (mg/g)	1.63	1.311
		K_1_ (g/mg min)	0.014	0.026
		R^2^	0.865	0.944
Pseudo-Second order	$\frac{\text{t}}{{q}_{t}}=\frac{1}{{K}_{2 }{q}_{e}^{2}}+\frac{t}{{q}_{e}}$	q_e_ (mg/g)	4.28	2.66
		K_2_ (g/mg min)	0.01	0.021
		R^2^	0.971	0.996
Intra-particle diffusion (IDP)	${q}_{t}={K}_{ad}{t}_{e}^{1/2}+\mathrm{C}$	K_ad_ (mg/g min^1/2^)	0.214	0.119
		C (mg/g)	1.05	1.012
		R^2^	0.944	0.938

### Isotherm study

**Figure 9:**
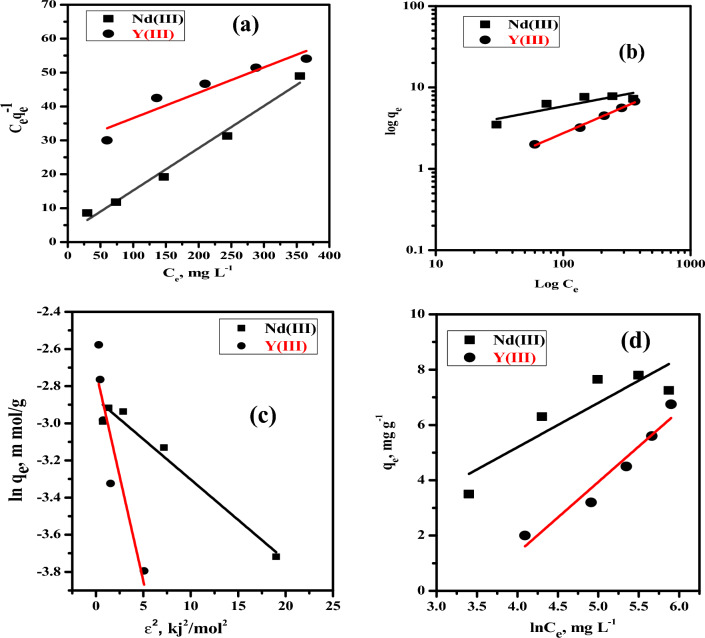
(**a**) Linear Langmuir isotherm plots; (**b**) Linear Freundlich isotherm plots; (**c**) linear Dubinin–Radushkevich (D-R) isotherm Plots, (**d**) linear Temkin isotherm plots for the sorption of of Nd(III), and Y(III) using nano-MgO/Ca-alginate beads from acidic nitrate medium.

Several isotherm models were used to match the experimental results. The Langmuir, Freundlich, Dubinin–Radushkevich (D-R), and Temkin models are the most commonly employed to describe the interaction behaviour of metal ions with the utilized nano-MgO/Ca-alginate beads (Fig. 9a–d), and Table 3 summarizes the calculated parameters and correlation coefficient (R^2^) provided by the models applied. The correlation coefficient (R^2^) value for the Langmuir model of Nd(III) (0.981) is higher than that of the investigated isotherm models. Furthermore, the maximum sorption capacity of Nd(III) was found to be 8.03 mg/g in the case of Langmuir and 8.19 mg/g in the case of the Dubinin–Radushkevich model, which were closer to the experimental results. This result demonstrates that the Langmuir model and Dubinin–Radushkevich are more appropriate for expressing experimental data. In the case of Y(III), the correlation coefficient (R^2^) value for the Freundlich model (0.994) is higher than that of the investigated isotherm models, and Dubinin–Radushkevich capacity (5.85 mg/g) is closer to experimental capacity This result shows that the Freundlich model and Dubinin–Radushkevich are more appropriate for describing experimental data.

**Table 3:** Calculated parameters of the linear Freundlich, Langmuir, D-R, and Temkin isotherm models for Nd(III) and Y(III) sorbed onto the nano-MgO/Ca-alginate beads.

Isotherm	Linear form equation	Parameters	Metal ions	
			Nd(III)	Y(III)
Langmuir	$\frac{{C}_{e}}{{q}_{e}}=\left(\frac{1}{{Q}_{o}b}\right)+\left(\frac{1}{{Q}_{o}}\right){C}_{e}$	Qo (mg/g)	8.03	13.37
		b (ml/mg)	0.05	0.003
		R_L_	0.167	0.77
		R^2^	0.981	0.878
Freundlich	$\mathrm{log}{q}_{e}=\mathrm{log}{K}_{f}+\frac{1}{n}\mathrm{log}{C}_{e}$	K_f_ (mg/g)	1.48	0.122
		N	3.34	1.48
		R^2^	0.698	0.994
Dubinin–Radushkevich (D-R)	$\mathrm{ln}{q}_{e}=\mathrm{ln}{q}_{m}-\beta {\varepsilon }^{2}$ $\varepsilon =RT\mathrm{ln}\left[1+\left(1/{C}_{e}\right)\right]$	*q*_*m*_ (mg/g)	8.19	5.85
		*Β*	0.043	0.226
		*R*^*2*^	0.957	0.822
Temkin	${q}_{e}=B\mathrm{ln}{K}_{t}+B\mathrm{ln}{C}_{e}$	*K*_*T*_	0.464	0.03
		*B*	1.607	2.569
		*R*^*2*^	0.724	0.927
q_exp_, mg/g			7.80	5.60

### Desorption investigations of loaded nano MgO/Ca-alginate beads with Nd(III) and Y(III)

The loaded nano-MgO/Ca-Alginate beads with Nd(III) and Y(III) was desorbed using different effective desorption reagents such as sulfamic acid, nitric acid, sulfuric acid, ammonium carbonate, and sodium acetate. The desorption experiments were studied at the same sorption conditions, including (contact time = 90 min, adsorbent weight of 0.1 g, at 25 ± 1 °C). The calculated percentage illustrated in Table 4, which indicates that the highest desorption of 42% and 51% for Nd(III) and Y(III) respectively, was achieved with 1 M sulfamic acid after one desorption cycle. Furthermore, the adsorption and desorption cycles for nano-MgO/Ca-alginate beads was investigated indicated that can be used for 4 adsorption cycles with S% of (65%, 45%, 35%, and 20%) for Nd and 3 adsorption cycles with S% of (40%, 35%, 15%) for Y.

**Table 4:** Values of desorption efficiency for Nd(III) and Y(III) after sorption on nano-MgO/Ca-alginate beads at pH = 2 and 25 ± 1°C.

Stripping agent, M	Conc., M	Nd(III)	Y(III)	S-ratio
				S_Y_/S_Nd_
Sulfamic acid	1	42	51	1.21
H_2_SO_4_	0.5	36.5	37	1.01
HNO_3_	0.5	35.5	39	1.09
(NH_4_)_2_CO_3_	0.5	33	42	1.27
sodium acetate	0.5	33	33.5	1.01

Furthermore, the separation feasibility from the desorption ratio (S-ratio) between the recovered metal ions was calculated by dividing their desorption percentages. The results show that the maximum (S-ratios) are 1.27 and 1.21 for S_Y_**/**S_Nd_ which were achieved with [ammonium carbonate] = 0.5 M, and [sulfamic acid] = 0.5 M respectively, Table 4.

### Comparison between nano-MgO/Ca-alginate beads with other reported materials

To emphasis on the adsorptive properties and efficiency of nano-MgO/Ca-alginate beads for sorption of Nd and Y from acidic medium a comparison report between Nd-Y/MgO/Ca-alginate system and others pervious adsorbent materials^55–61^ were studied. The results were given in Table 5, shows the maximum adsorption capacity (Q_0_) of nano-MgO/Ca-alginate beads was 7.85 mg/g for Nd which is more efficient than some adsorbent such as (clay minerals and Alkyl phosphinic acid resin) and closed efficiency to others (Fe_3_O_4_@SiO_2_@polyaniline-graphene oxide and Fe_3_O_4_@TiO_2_@P_2_0_4_ nanoparticle). On the other hand the maximum adsorption capacity (Q_0_) of nano-MgO/Ca-alginate beads for Y was 5.6 mg/g which is more efficient than (Carbon black derived from recycled tires, Kaolin and Kaolinite). The previous comparisons data proved the efficiently of the used MgO/Ca-alginate beads for sorption of Nd and Y from low acidic medium.

**Table 5:** Comparison study between nano-MgO/Ca-alginate beads and other reported materials for sorption of Nd and Y from acidic medium.

Metal ion	Adsorbent	Q_o_, mg/g	References
Nd(III)	MgO/Ca-alginate beads	7.85	This work
	porous three-dimensional graphene oxide-corn zein composites	9.7	^55^
	Clay minerals	1.58	^56^
	Alkyl phosphinic acid resin	2.02	^57^
	Fe_3_O_4_@TiO_2_@P_2_0_4_ nanoparticles	8.66	^58^
	Fe_3_O_4_@SiO_2_@polyaniline-graphene oxide	8.50	^59^
Y(III)	MgO/Ca-alginate beads	5.60	This work
	Kaolinite	2.56	^60^
	Carbon black derived from recycled tires	0.608	^61^
	Magnetic silica hybrid material with P507	10.32	^62^
	Nano maghemite	13.5	^63^

## Conclusions

The experimental batch conditions for sorption of Nd-Y onto nano-MgO/Ca-alginate beads were set up as experimental conditions (time = 90 min, T = 25 °C, and V/m = 0.05 L/g, and pH = 2). Nano-MgO/Ca-alginate beads were successfully used for the recovery of Nd(III) and Y(III) from aqueous acidic nitrate solution at pH = 2. The calculated maximum capacity of nano-MgO/Ca-alginate beads is 12.6 and 7.85 mg/g for Nd and Y respectively at the optimum selected sorption factors. Thermodynamic parameters show that the sorption of Nd and Yon nano-MgO/Ca-alginate beads is endothermic process. The maximum stripping of 42% and 51% for Nd(III) and Y(III) respectively, was achieved with 1 M sulfmic acid after one desorption cycle. The highest separation ratio was found to be 1.27 and 1.21 which were achieved with 1 M and 0.5 M of ammonium carbonate and sulfamic acid respectively. These kinetics investigations results show that the sorption process of Nd(III) and Y(III) on Mgo/Cal-alginate nano-beads is performed by the chemisorption process. The comparison study proved the efficiency of nano-MgO/Ca-alginate beads as prospective adsorbent for Nd and Y from low acidic medium. In the future research work, a feasibility study will be done to prepare the material (nano-MgO/Ca-alginate beads) and take advantage of its applications in the separation processes of rare earth elements into groups (light, medium, and heavy) and conduct an industrial application to separate them from some raw materials such as Egyptian monazite and some wastes resulting from various industries such as electronics, glass, and others.

## Data Availability

All data generated or analyzed during this study are included in this published article [and its supplementary information files].
